# Study on the effects and mechanisms of rhythmic auditory stimulation on freezing of gait in Parkinson’s disease: investigation based on functional near-infrared spectroscopy (fNIRS) technology

**DOI:** 10.3389/fneur.2026.1802799

**Published:** 2026-05-13

**Authors:** Lingyu Sheng, Ziyao Zhang, Guiyun Cui, Jie Xiang

**Affiliations:** 1 College of Medical Technology, Xuzhou Medical University, Xuzhou, China; 2 The Second Clinical Medical College, Xuzhou Medical University, Xuzhou, China

**Keywords:** auditory cues, freezing of gait, gait, near-infrared functional brain imaging, Parkinson’s disease

## Abstract

**Introduction:**

Freezing of gait (FOG) in Parkinson’s disease (PD) patients is a critical determinant of motor impairment and fall risk. Rhythmic auditory stimulation (RAS) has been shown to ameliorate FOG symptoms, although the underlying neurophysiological mechanisms are not fully understood.

**Objectives:**

This research employed functional near-infrared spectroscopy (fNIRS) to analyze three RAS—two external auditory cueing strategies (beat perception and music therapy) and one internal cueing method (mental beat imagery)—to examine gait parameters and cortical activation patterns in Parkinson’s disease patients with freezing of gait (PD + FOG) compared to healthy controls (HC). The study also sought to evaluate the therapeutic effectiveness of these rhythm-based interventions in alleviating FOG episodes.

**Methods:**

Twenty-eight patients with PD + FOG and twenty-eight age-matched HC were enrolled in the study. Gait analysis was performed during narrow corridor ambulation, designed to induce freezing episodes, and during straight-line walking in an open environment. fNIRS was employed to measure fluctuations in oxygenated hemoglobin (HbO_2_) and deoxygenated hemoglobin (HHb) concentrations, serving as indicators of cortical activation. Regions of interest (ROIs) included the prefrontal cortex (PFC), premotor cortex (PMC), and temporal cortex (TLC). Intracortical functional connectivity during each locomotor task was evaluated through correlation analyses of HbO_2_ signals between the ROIs.

**Results:**

PD + FOG patients exhibited gait disturbances characterized by reduced gait velocity and stride length, along with increased mediolateral postural sway during freezing episodes. Neuroimaging indicated significantly decreased activation in the primary somatosensory cortex (S1), PMC, and PFC compared to healthy controls, despite elevated intracortical connectivity involving indirect corticospinal pathways. Differential responses to RAS interventions were noted: rhythmic auditory cues enhanced connectivity between TLC and PMC; music therapy significantly improved PFC intrinsic connectivity; whereas imagined auditory cues potentially impaired sensorimotor integration due to excessive reliance on internal cognitive mechanisms.

**Conclusion:**

Various RAS pathways can ameliorate freezing of gait symptoms by selectively modulating functional connectivity between the prefrontal cortex and sensorimotor circuits. The underlying neural mechanisms may largely involve neural entrainment and reorganization of brain networks. These results offer vital empirical insights into the pathophysiology of gait freezing and provide a preliminary theoretical basis for designing precision neuromodulation interventions.

## 1 Introduction

Freezing of gait (FOG) is a highly prevalent motor symptom in advanced Parkinson’s disease (PD), significantly contributing to functional impairment and increased fall risk among patients. FOG is characterized by a transient inability to initiate or continue stepping, often described as feet appearing “glued” to the ground, hindering voluntary locomotion (1, 2). It demonstrates pronounced context dependence, frequently occurring during gait initiation (such as transitioning from sitting to walking), turning, navigating constricted environments (like doorways and hallways), and approaching target locations (such as chairs or dining tables) (3). This episodic motor phenomenon not only elevates the likelihood of falls but also promotes activity avoidance behaviors, which can lead to secondary complications including reduced mobility and muscle atrophy, thereby perpetuating a detrimental cycle.

Among therapeutic interventions for freezing of gait, Rhythmic Auditory Stimulation (RAS) has demonstrated promising clinical potential due to its non-invasive and user-friendly characteristics (4). RAS offers external compensatory cues by delivering rhythmic auditory stimuli—such as music or rhythmic sounds—that facilitate neural network integration and enhance coordination between postural stability and locomotor movements, thereby decreasing the incidence of FOG episodes (5). Empirical evidence confirms that gait-specific RAS interventions significantly improve gait velocity and cadence in Parkinson’s disease patients (6). Additionally, external rhythmic cues can modulate motor timing, markedly influencing FOG frequency, duration, and gait fluidity (7). Clinically, RAS primarily utilizes external auditory stimuli, including beat-based listening (e.g., fixed-frequency auditory pulses) and music-based interventions that leverage rhythmic musical features. Studies indicate that music characterized by high rhythmicity provides more accurate motor synchronization cues and elicits greater spontaneous movement. The underlying mechanism involves the coupling of sensory input with motor output, enabling effective musical rhythm entrainment to optimize gait rehabilitation outcomes (8). Internal cueing strategies, such as patients imagining rhythmic walking, may enhance subjective engagement and gait performance; however, current research findings are inconclusive, and consensus has yet to be established (9, 10).

Currently, numerous hypotheses and computational models have been proposed within the neuroscientific domain to elucidate the pathophysiological mechanisms underlying FOG, including abnormal gait pattern generation, autonomic dysregulation, perceptual deficits, maladaptive coupling between postural control and locomotor circuits, and executive dysfunction (11). Evidence suggests that the RAS may enhance motor function by compensating for disrupted neural pathways in PD patients (12). Considering that the basal ganglia are critical for internal timing and rhythmic perception, their dysfunction in PD impairs these processes, leading to a loss of rhythmicity in temporally coordinated movements such as gait, thereby triggering FOG episodes. The RAS potentially mitigates this impairment by engaging cerebellar circuits, thus enhancing gait stability (13). An additional prominent mechanism involves rhythm entrainment and oscillatory coupling (14), where entrainment refers to the periodic synchronization of neural oscillations with external rhythmic stimuli. The RAS may induce entrainment of cortical and subcortical oscillations, aligning neural activity with external cues to reduce FOG occurrences (15). Slow-frequency oscillations facilitate widespread neuronal recruitment across extensive brain networks and can couple with local fast-frequency oscillations, enabling cross-frequency interactions within and between auditory and non-auditory cortical regions, thereby promoting neural coherence (16, 17). Furthermore, to our knowledge, the neurophysiological characteristics of brain dysfunction in PD patients during real-world gait activities, particularly within the frontotemporal cortex, remain insufficiently investigated (18).

In the investigation of neurophysiological processes, functional near-infrared spectroscopy (fNIRS) serves as an innovative, non-invasive neuroimaging modality. By measuring fluctuations in near-infrared light absorption within cerebral tissue, it quantifies variations in oxygenated hemoglobin (HbO_2_) and deoxygenated hemoglobin (HHb), thereby indirectly assessing hemodynamic responses and cortical functional states. Additionally, signals from distinct brain regions are analyzed to compute functional connectivity (FC), elucidating interregional neural network interactions. This modality offers advantages such as moderate spatial and temporal resolution, high resistance to motion artifacts, and the capacity for data acquisition during naturalistic behaviors, rendering it a suitable tool for examining cortical activation patterns during RAS interventions (19, 20).

Based on this, the present study hypothesizes that external auditory stimulation enhances functional connectivity between motor and auditory regions, thereby improving gait; in contrast, internal auditory stimulation may increase the cognitive load on these brain regions, resulting in a less pronounced effect on gait improvement. Accordingly, this study aims to use three types of rhythmic auditory stimulation—auditory beat stimulation (RAS-B), musical stimulation (RAS-M), and internal cues via imagery of rhythmic beats (RAS-I)—in conjunction with fNIRS to compare and analyze cortical activation patterns and neural connectivity differences between Parkinson’s disease patients with freezing of gait (PD + FOG group) and healthy controls (HC) during intervention. The research seeks to elucidate the neural mechanisms underlying gait improvement through varying RAS modalities and identify the key cortical neural pathways involved in RAS-based therapy.

## 2 Materials and methods

### 2.1 Participants

This study employed a two-stage design, and sample size estimation was performed using G*Power 3.1 software, with calculations conducted separately for each primary research objective. A small pilot study was conducted prior to formal enrollment to validate the feasibility of the intervention protocol and unify experimental operating procedures. Considering the limited sample of the pilot study, the effect sizes adopted in this study were determined based on previously published relevant studies in the field (5, 21, 22). For between-group comparisons, this study utilized the independent samples t-test for statistical analysis. The effect size for the primary outcome measure (walking speed) was set at *d* = 0.8, with a significance level of *α* = 0.05 and a power (1-*β*) of 0.8. Calculations indicated that at least 21 participants were required per group. For the comparison of intervention effects, a repeated-measures analysis of variance (ANOVA) was employed. With an effect size of *f* = 0.4, *α* = 0.05, power (1-*β*) = 0.8, four measurement points, and an intra-group correlation coefficient of 0.5, the calculation indicated that the PD + FOG group required a total sample size of 20 participants. We adopted the larger sample size requirement to ensure sufficient statistical power for all analyses. Combining the results of these two estimates, this study used the 21 subjects required for between-group comparisons as the baseline. Additionally, considering a potential dropout rate of approximately 15% during the study, it was ultimately determined that 28 subjects would be enrolled in each group, for a total sample size of 56.

This research enrolled 28 Parkinson’s disease patients with PD + FOG and 28 HC from Xuzhou Medical University Affiliated Hospital, comprising a total sample size of 56 subjects. All participants provided written informed consent prior to data collection. The study received ethical approval (Ethics Approval Number: XYFY2025-KL351-01).

Inclusion criteria: (1) Patients diagnosed with Parkinson’s disease at Xuzhou Medical University Affiliated Hospital based on the 2015 Movement Disorder Society (MDS) clinical diagnostic guidelines (23); (2) age range 55–80 years; (3) Hoehn-Yahr (H-Y) stages II-III; (4) presence of gait freezing confirmed by the Freezing of Gait Questionnaire [FOG-Q (24)]; (5) ability to ambulate independently without assistive devices; (6) intact auditory and visual functions, evidenced by accurate performance on the whisper test and a Snellen visual acuity of at least 6/12.

Exclusion criteria: (1) secondary Parkinsonism, Parkinsonian syndromes, or other pre-existing neurological conditions such as epilepsy or encephalitis; (2) significant cognitive deficits indicated by a Mini-Mental State Examination [MMSE (25)] score below 24; (3) hearing impairment or deafness; (4) comorbid conditions affecting gait, including musculoskeletal disorders, visual impairments, vestibular dysfunction, or severe orthostatic hypotension; (5) inability to sustain a single activity continuously for less than 2 min; (6) history of deep brain stimulation surgery.

The following assessments were conducted during the patient’s “OFF” phase. The “OFF” state was defined as a period of motor symptom control, typically within 3 h after levodopa administration, characterized by improved motor function. Demographic data, including age, gender, education level, age at PD onset, and disease duration, were collected via structured interviews. Disease severity was evaluated using the H-Y staging system. Motor symptom severity was assessed with the MDS-UPDRS Part III [Movement Disorder Society-Unified Parkinson’s Disease Rating Scale Part III (26)]. FOG was evaluated using the FOG-Q. Additionally, demographic information of HCs, such as age, gender, and education level, was recorded, and cognitive function was assessed using the MMSE.

### 2.2 Experimental task

**Figure 1:**
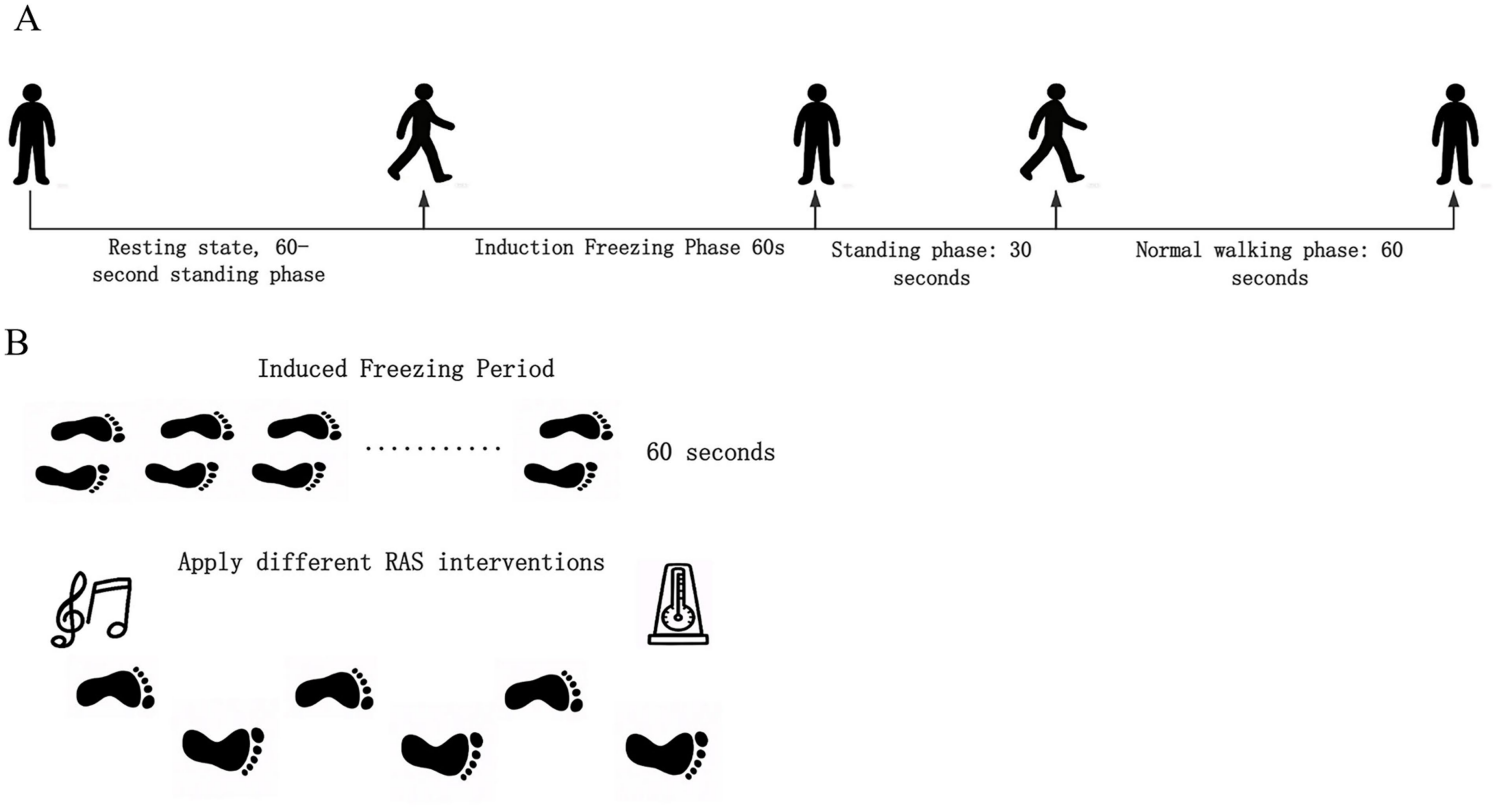
Schematic of the experimental paradigm. **(A)** The overall experimental procedure, including two distinct task phases. **(B)** The process of freezing-of-gait induction and the pre-post comparisons under beat and music auditory interventions.

The experimental walking protocol was as follows: participants initially stood quietly for 1 min, followed by the sequential execution of two core tasks: (1) walking and turning within a narrow corridor to induce FOG and maximize their occurrence; (2) straight-line walking in an open area to serve as the normal gait phase, during which no freezing episodes occurred. The complete experimental paradigm comprised: 1 min of static standing, 1 min of freezing-induction walking, 30 s of static rest, 1 min of normal walking, and 1 min of static standing post-activity (see Figures 1A,B for the schematic diagram). A 10-s preparatory period was applied before the initial standing phase to ensure participants reached a relaxed state. The interleaved static intervals helped maintain consistent attention and cognitive load throughout the experiment. The final 30-s standing period post-task served as a post-baseline to correct for data drift. Considering that freezing phenomena were more likely during initial gait initiation in patients with PD, the protocol first induced FOG before proceeding with normal walking.

Prior to the experiment, participants underwent comprehensive baseline assessments, including motor function evaluation, cognitive assessment, and audiovisual capability testing. During the procedure, subjects were equipped with fNIRS sensors to monitor cortical activation patterns and brain network connectivity. Gait analysis was performed using a gait and posture analysis system (Right Gait and Posture) to record parameters such as stride length, gait velocity, and cadence. Additionally, video recordings were used to document the frequency of FOG.

Intervention measures utilized three RAS modalities: RAS-B, RAS-M and RAS-I. RAS-B employed metronome stimuli set at a tempo 10% faster than the participant’s normal gait cadence (27); all RAS interventions required participants to synchronize their steps with the metronome or music rhythm during ambulation. RAS-M selected musical excerpts based on individual preferences from Return from Target Practice and Hero’s Song, with tempo adjusted using a speed controller to match the prescribed metronome frequency, and volume set to a comfortable perceptual level for the participant. RAS-I involved the participant initially listening to the rhythm, then internally maintaining that rhythm pattern during walking.

After each intervention condition, subjects were provided approximately a 30-min rest period to prevent fatigue from impacting subsequent experimental outcomes. Participants were blinded to the intervention sequence, and the order of auditory cues (RAS-B, RAS-M) was randomized to eliminate potential confounding effects of fatigue or learning. Since RAS-I required manual guidance, it was scheduled as the final condition. During the experiment, fNIRS data and gait parameters were collected under various intervention conditions, with subjects instructed to minimize unnecessary head movements and swallowing. The laboratory environment maintained silence to minimize disturbances and reduce cortical activity unrelated to the task.

### 2.3 fNIRS

**Figure 2:**
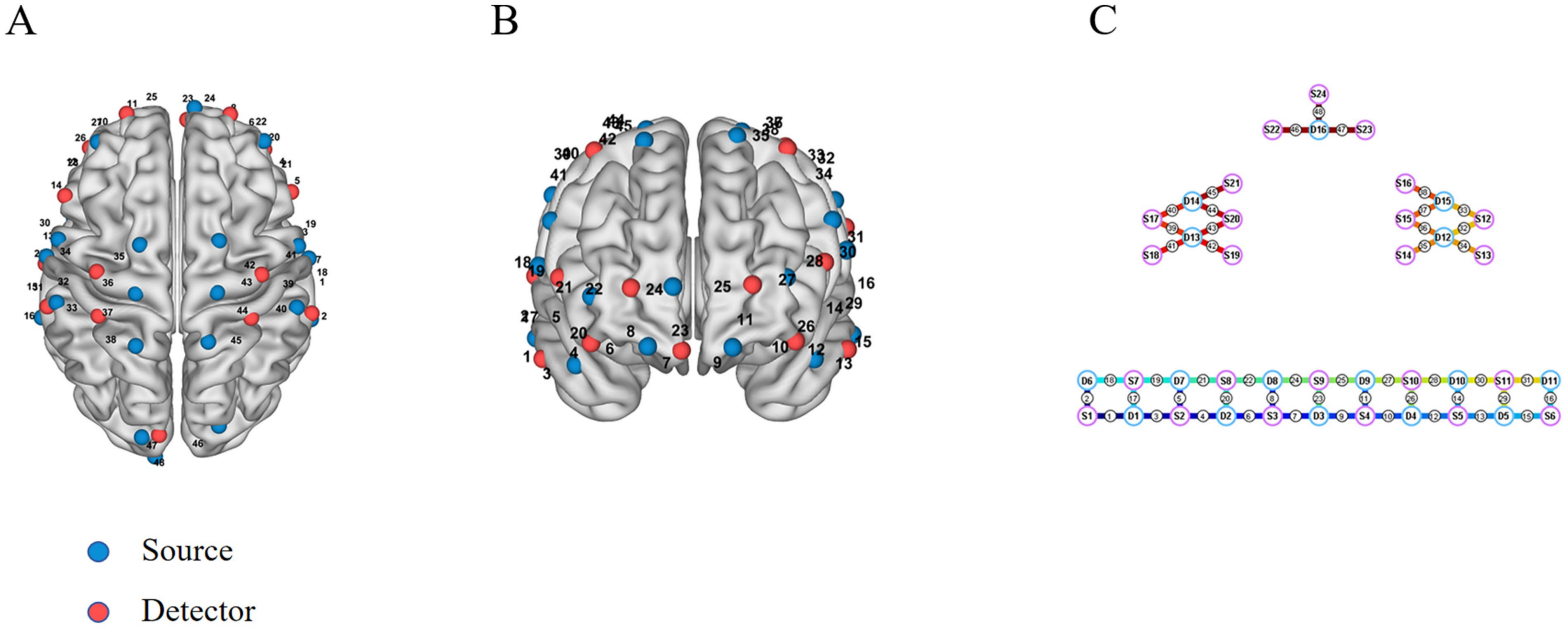
The corresponding positions of the fNIRS probe array on the cerebral cortex. Panels **(A,B)** show the top and frontal views of probe distribution over the cerebral cortex, respectively, and panel **(C)** provides a two-dimensional schematic for intuitive visualization of the channel layout.

This investigation utilized a portable fNIRS system (Nirsmart; Danyang Huichuang Medical Equipment Co., Ltd., Danyang, China). The device quantified the optical absorption of HbO_2_ and HHb using near-infrared wavelengths of 730 nm and 850 nm at a sampling frequency of 11 Hz. A total of 48 fNIRS optodes (comprising 24 light sources and 16 detectors) covered cortical regions including the frontal, parietal, and temporal lobes. Regions of interest (ROIs) encompassed the premotor cortex (PMC) corresponding to channels 19, 30, 34, 35, 36, 41, 42, 43; the primary somatosensory cortex (S1) corresponding to channels 31, 32, 33, 39, 40; the primary motor cortex (M1) corresponding to channels 37, 44; the prefrontal cortex (PFC) corresponding to channels 4, 6, 7, 8, 9, 10, 11, 12, 22, 23, 24, 25, 26, 27; and the temporal cortex (TLC) corresponding to channels 1, 2, 3, 5, 13, 15, 16, 17, 18, 29. For precise localization of the targeted cerebral regions (refer to Figures 2A–C). The system employed three-dimensional spatial localization principles aligned with the international 10–20 electrode placement system (28), utilizing anatomical landmarks such as the nasion, external occipital protuberance, and preauricular points. ROI placement was calibrated according to standardized cap sizes (XS to L) to accommodate variations in head circumference. The inter-optode distance was set at 3 cm. Participants wore a black head cap to reduce ambient light interference with optical signals. Pre-experimental signal intensity verification was conducted to ensure data integrity.

### 2.4 Data analysis

Considering that HbO₂ demonstrated higher sensitivity to cerebral perfusion alterations compared to HHb (29), this investigation utilized the HbO₂ signal as the neurovascular activation biomarker for subsequent neuroimaging analysis. The preprocessed fNIRS data were segmented according to experimental epochs: (1) Baseline resting state (0—60 s, during which subjects remained stationary); (2) Induced freezing episode (62.5—120 s following task onset); (3) Normal ambulation phase (152.5—210 s post-task initiation).

First, fNIRS data were preprocessed using a standardized pipeline. Signal quality assessment was first performed by calculating the signal-to-noise ratio (SNR) for each channel based on the coefficient of variation (CV), and channels with low signal quality (CV > 14%) were excluded from subsequent analysis (30). Optical intensity signals were then converted into changes in HbO₂ and HHb concentrations according to the modified Beer–Lambert law. Motion artifacts caused by head movements and swallowing were removed using a combined approach of standard deviation and cubic spline interpolation with parameter settings of STDEV-thresh = 6.0 and AMP-thresh = 0.5 (31). A Butterworth bandpass filter with a frequency range of 0.01—0.2 Hz was applied to reduce physiological noise including cardiac pulsations, respiratory cycles, and low-frequency baseline drift (32). The differential path length factor (DPF) was set to 6.0 (33), and block averages were calculated within the time windows corresponding to each experimental condition. Baseline correction was performed using the 5 s immediately before walking as the baseline interval, and HbO₂ signals from 3 to 60 s during walking were extracted to accommodate the typical hemodynamic response latency, which occurs 1—2 s after neural activation and peaks at 4—7 s (34). Finally, the mean change in HbO₂ concentration relative to baseline was determined for each task condition by subtracting the mean baseline value from the mean value obtained during the task period.

Data processing and statistical analyses were all performed using SPSS software version 26.0, with the significance level for all statistical tests uniformly set at *p* < 0.05. The present study conducted comparative analyses focusing on the following four core aspects, with detailed procedures as follows: First, comparisons of general demographic data and gait parameters were performed. Normality tests were conducted for all data prior to analysis; parametric tests such as independent-samples t-tests and ANOVA were used for normally distributed continuous data, while non-parametric tests including Mann–Whitney *U* test and Kruskal–Wallis *H* test were adopted for non-normally distributed continuous data to ensure the rationality of statistical method selection. Second, comparisons of baseline brain activation were carried out: for each measurement channel of fNIRS, the mean HbO₂ concentration values of the HC group and the PD + FOG group without intervention were calculated separately during the resting state, freezing-induction walking phase, and normal walking phase. Independent-samples *t*-tests were used to compare the mean HbO₂ values of each channel between the two groups one by one, so as to clarify the differences in brain region activation between the two groups at different task phases. Meanwhile, brain activation maps of each group were generated to intuitively present the spatial distribution of activation differences. Given the large number of fNIRS measurement channels, the Benjamini–Hochberg (BH) procedure was applied for false discovery rate (FDR) correction to control the false positive rate associated with multiple comparisons across channels, and only channels that passed the correction were considered to have significant inter-group differences. Third, comparisons of RAS intervention effects were conducted: for the PD + FOG group, one-way repeated-measures ANOVA was used to compare the mean HbO₂ concentration values of each fNIRS channel under three different RAS intervention conditions (listening to beats, listening to music, and imagining beats), so as to analyze the impact of different intervention methods on brain region activation. If the repeated-measures ANOVA showed a significant main effect, Fisher’s least significant difference (LSD) post-hoc test was further used for pairwise comparisons to clarify the differences in brain region activation between any two intervention conditions; FDR correction was also applied for multiple comparisons across channels in this part to control false positives. Fourth, functional connectivity (FC) analysis was performed: based on the HbO₂ concentration changes of each fNIRS channel, Pearson correlation coefficients were used to calculate the functional connectivity strength between each pair of regions of interest (ROIs), between channels, and within brain regions one by one, forming a functional connectivity matrix. To control the error of multiple comparisons, FDR correction was applied to the correlation coefficients of all functional connectivity strengths. Subsequently, repeated-measures ANOVA was used to compare the functional connectivity differences within groups (among different intervention conditions of the PD + FOG group) and between groups (between the HC group and the PD + FOG group), covering all functional connectivity pairs between channels, between brain regions, and within brain regions. Pairwise comparisons were performed between each RAS condition and the no-RAS baseline. For groups or conditions with significant differences, LSD post-hoc test was further used to clarify the specific source of the differences.

## 3 Results

### 3.1 Demographic and clinical characteristics

This study included a total of 56 subjects, comprising 28 PD + FOG and 28 HC. As shown in Table 1, there were no significant differences between the PD + FOG group and the HC group across all measured variables.

**Table 1:** Baseline demographic characteristics of participants.

Basic information	HC (*N* = 28)	PD + FOG (*N* = 28)	Statistical value	*p*-value
Gender (Male/Female, e.g.)	13/15	16/12	−0.795	0.427
Age (years)	67.71 ± 4.86	69.86 ± 6.264	1.430	0.158
Height (m)	1.65 (1.56, 1.75)	1.65(1.57, 1.71)	−0.164	0.870
Weight (kg)	62.50 (57.25, 78.00)	65.00 (60.00, 78.75)	−0.320	0.749
Drinking History (%)	12 (42.9)	15 (53.6)	−0.280	0.780
Smoking History (%)	10 (35.7)	9 (32.1)	−0.795	0.427
BMI (kg/m^2^)	24.59 ± 1.95	24.79 ± 3.00	0.286	0.776
Years of Education (years)	5.00 (2.00, 8.75)	8.00 (1.00, 9.00)	−1.028	0.304
MMSE	24.50 (23.25, 26.00)	24.50 (21.25, 27.00)	−0.661	0.508
UPDRS-III	\	37.50 (28.25, 45.25)	\	\
H-Y stage (II–III)	\	3.00 (2.00, 3.00)	\	\
FOG-Q (0–24)	\	10.00 (8.00, 12.00)	\	\
Disease duration	\	6.93 ± 2.75	\	\

For normally distributed quantitative data, results are expressed as mean ± standard deviation (SD), and comparisons between groups were performed using the independent samples *t*-test. For non-normally distributed data, results are presented as median (interquartile range, IQR), and comparisons between groups were conducted using the Mann–Whitney *U* test. Categorical variables were analyzed using the chi-square (*χ*^2^) test.

### 3.2 Comparison of gait parameters

#### 3.2.1 Comparison of gait parameters between freezing episodes and normal walking periods

**Figure 3:**
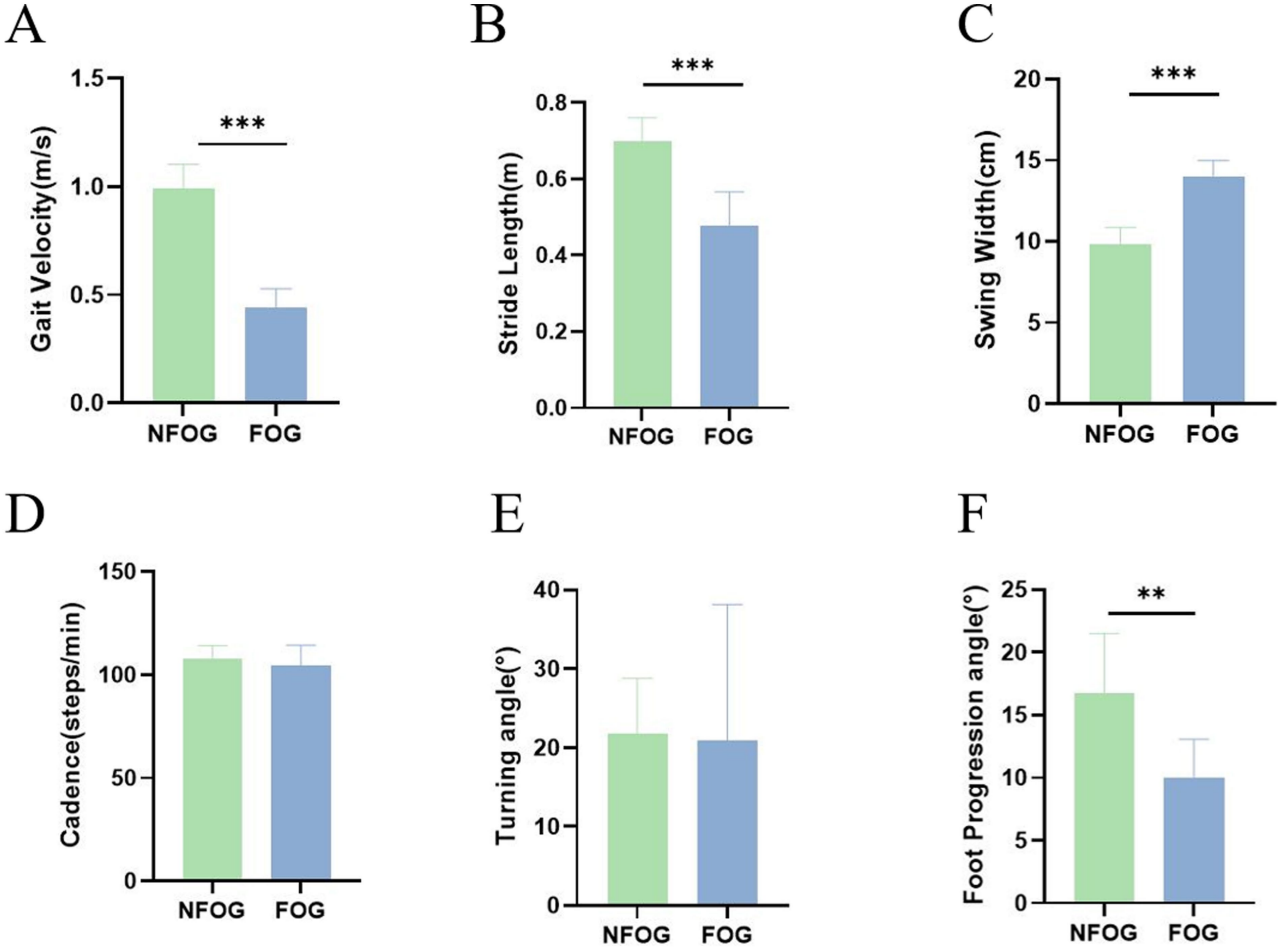
**(A–F)** Comparison of gait parameters between the freezing-induction period and normal walking phase in PD patients. *p < 0.05, **p < 0.01, ***p < 0.001.

During normal gait cycles, patients with PD + FOG exhibited significantly higher cadence and longer stride length compared to those during the freezing-induction period, with a notable reduction in swing width. Statistical analysis demonstrated that the differences in gait parameters—cadence, stride length, foot progression angle, and swing width—between the freezing-induction period and normal walking were statistically significant, indicating that the freezing-induction period is characterized by decreased gait velocity, reduced stride length, and increased swing width. Detailed data are presented in Figures 3A–F.

#### 3.2.2 Comparison of gait parameters during freezing episodes across different intervention methods

**Figure 4:**
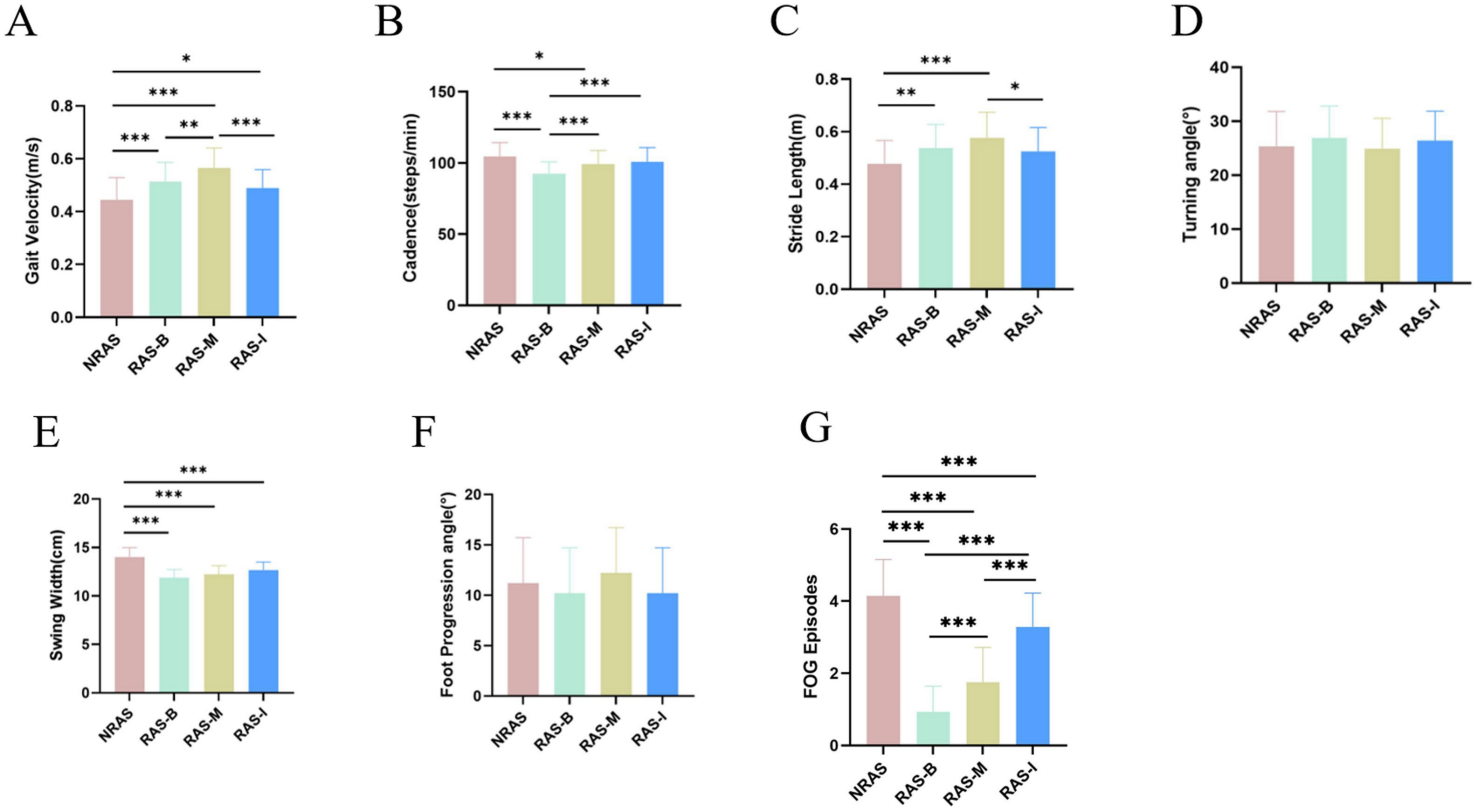
**(A–G)** Comparison of gait parameters across different intervention groups. *p < 0.05, **p < 0.01, ***p < 0.001.

NRAS, RAS-B, RAS-M, and RAS-I each demonstrated slight increases in cadence and stride length compared to the no-intervention group, along with a modest reduction in swing width. Additionally, the occurrence of freezing gait during the freezing-induction period significantly decreased. As shown in Figures 4A–G, RAS-B and RAS-M interventions exhibited more pronounced improvements, whereas the effects of RAS-I intervention were not statistically significant.

### 3.3 fNIRS data

#### 3.3.1 Brain activation characteristics

##### 3.3.1.1 Comparison of brain activation between HC group and PD non-intervention

**Figure 5:**
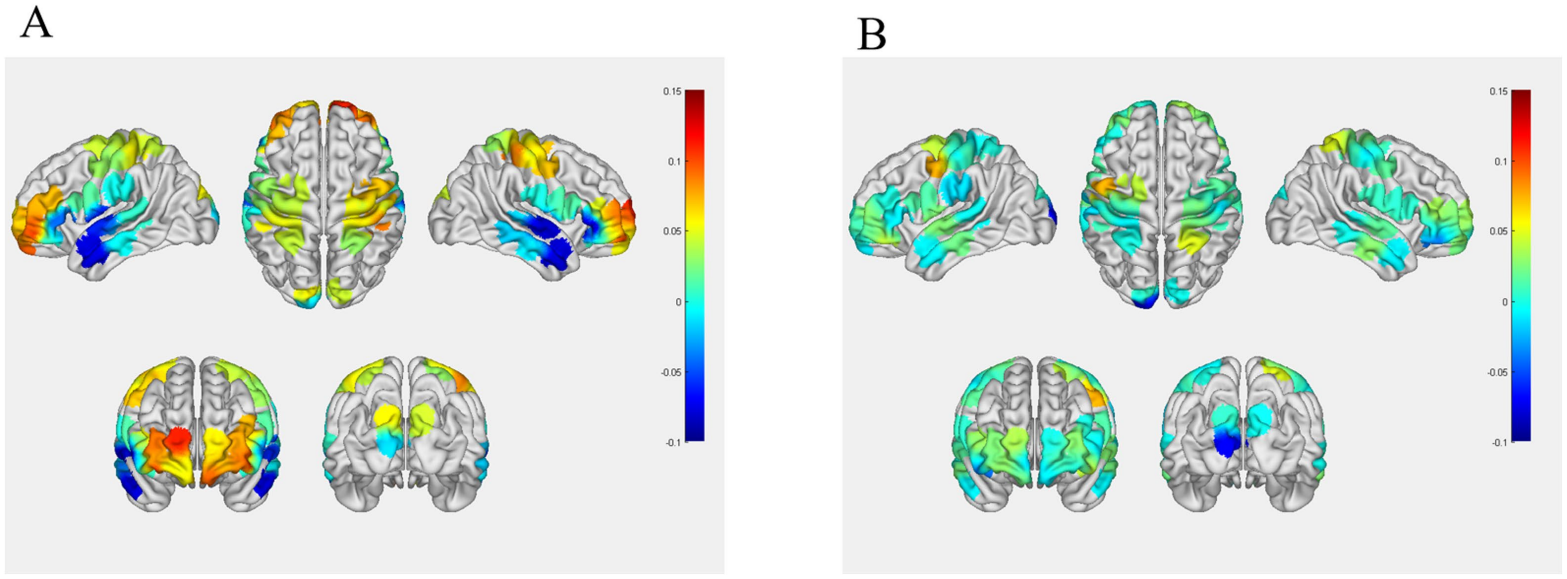
**(A)** Mean HbO_2_ levels in healthy controls; **(B)** Mean HbO_2_ levels in the PD control group. The colormap on the right side of the figure indicates that brain activation is more significant as the color approaches red.

Figure 5 clearly shows that brain activation was significantly higher in the HC group than in the PD group.

Subsequent statistical analysis revealed that during the freezing episodes, the HC group exhibited significantly elevated HbO_2_ levels in the S1 (*p* = 0.007, Cohen’s *d =* −0.88), M1 (*p* = 0.003, Cohen’s *d =* −0.91), and PFC (*p* = 0.006, Cohen’s *d =* −0.91) regions compared to the PD group without intervention; no significant differences were observed in other brain regions between the groups (refer to Table 2 for detailed results).

**Table 2:** Comparison of HbO_2_ levels between healthy individuals and the non-intervention group during the freezing period.

Time period	ROI	HC	PD + FOG	Statistical value	Cohen’s *d*	*p* -value
Induced freezing period	S1	0.036 ± 0.060	−0.011 ± 0.067	−2.796	−0.88	0.007**
	PMC	0.025 ± 0.047	0.009 ± 0.049	−1.214	−0.38	0.23
	M1	0.043 ± 0.058	−0.007 ± 0.065	−3.066	−0.97	0.003**
	PFC	0.034 ± 0.045	−0.013 ± 0.075	−2.878	−0.91	0.006**
	TLC	−0.000 ± 0.071	0.021 ± 0.079	1.055	0.33	0.296

*p < 0.05, **p < 0.01, ***p < 0.001.

##### 3.3.1.2 Comparison of brain activation across intervention groups

Repeated measures analysis revealed no statistically significant differences in brain activation levels among the four groups (*p* < 0.05), except for TLC (Channel 1: *p* = 0.021, partial *η^2^ =* 0.54; Channel 2: *p* = 0.019, partial *η^2^ =* 0.54) (refer to Table 3 for detailed neurophysiological data).

**Table 3:** Comparison of HbO_2_ levels in TLC channels across different intervention groups.

CH	None	RAS-B	RAS-M	RAS-I	Statistical value	Partial *η^2^*	*p* -value	*p* -value (*post hoc*)		
								None vs. RAS-B	RAS-B vs. RAS-M	RAS-B vs. RAS-I
CH1	0.044 ± 0.064	−0.011 ± 0.085	0.053 ± 0.084	0.050 ± 0.084	3.444	0.54	0.021*	0.012*	0.008**	0.032*
CH2	0.016 ± 0.084	−0.021 ± 0.070	0.031 ± 0.059	0.032 ± 0.061	3.496	0.54	0.019*	–	0.002**	0.007**

*p < 0.05, **p < 0.01, ***p < 0.001.

#### 3.3.2 Functional connectivity (FC) characteristics

##### 3.3.2.1 Comparison between HC group and PD group without intervention

**Figure 6:**
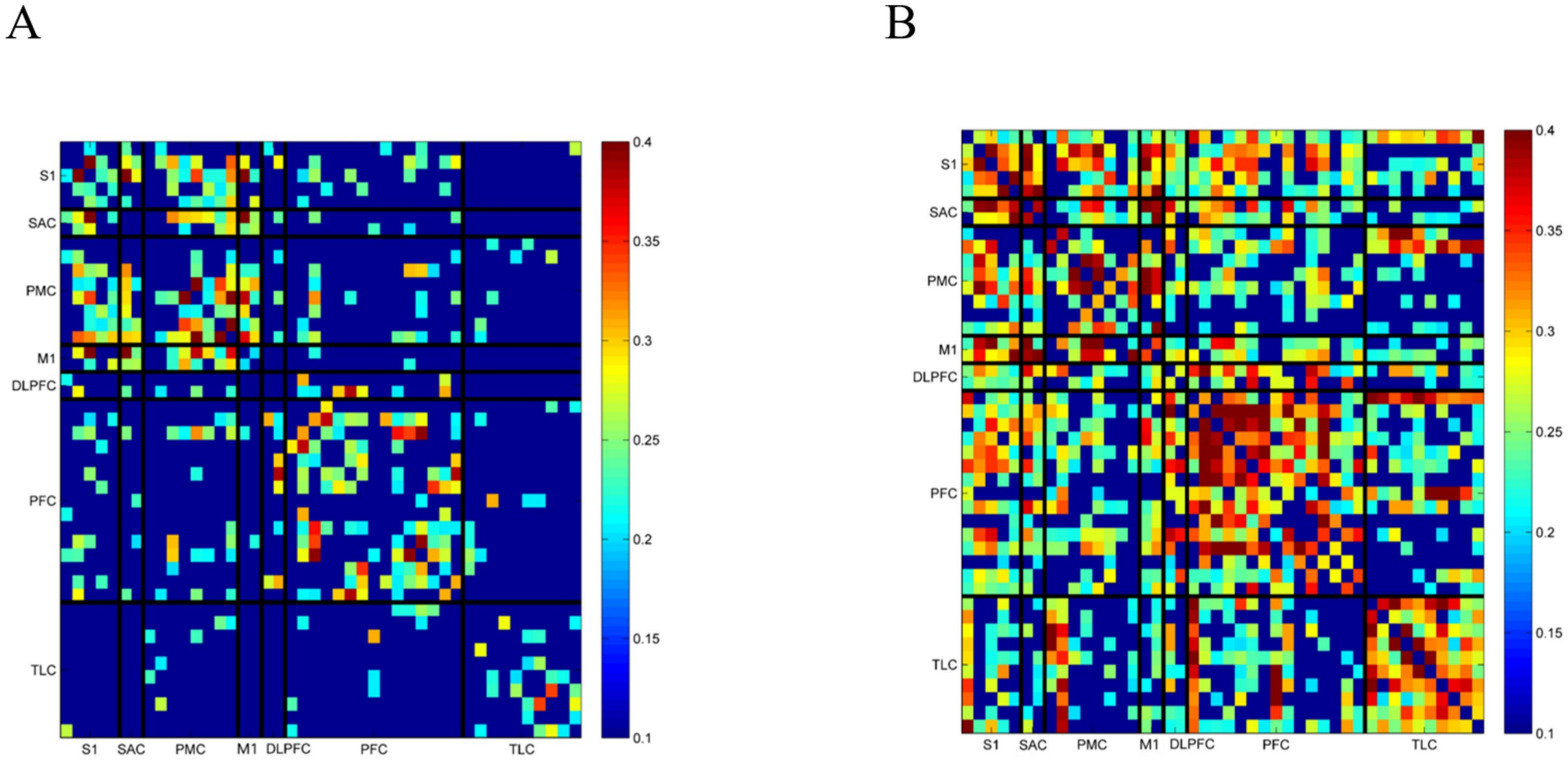
Comparison of functional connectivity between healthy controls and the untreated PD group. **(A)** Healthy controls; **(B)** Untreated PD patients. Different color blocks represent correlation coefficients, with warmer colors (especially red) indicating larger values.

To further investigate the differences in functional connectivity of the brain under various RAS conditions, the Pearson correlation coefficients between channels were computed to construct FC matrices. As shown in Figures 6A,B, the correlation coefficient matrices compare the channel-to-channel connectivity between the HC group and the PD + FOG group.

Table 4 presents statistically significant findings on functional connectivity between ROI pairs in the HC group and the PD group without intervention. Overall, the mean functional connectivity values in the PD no-intervention group are higher than those in the HC group, with differences reaching statistical significance (*p* < 0.05). Notably, the differences are especially pronounced within the prefrontal cortex-related networks.

**Table 4:** Comparison of functional connectivity in key brain regions between the HC group and the PD + FOG.

ROI or Ch name	HC	PD + FOG	Statistical value	Cohen’s *d*	*p*-value
S1-PFC	0.124 ± 0.112	0.239 ± 0.171	−2.968	0.80	0.013*
S1-TLC	0.047 ± 0.124	0.202 ± 0.191	−3.588	0.96	0.003**
PMC-TLC	0.054 ± 0.106	0.187 ± 0.152	−3.806	1.02	0.003**
M1-PFC	0.109 ± 0.141	0.240 ± 0.195	−2.874	0.77	0.014*
M1-TLC	0.024 ± 0.150	0.198 ± 0.200	−3.687	0.98	0.003**
PFC-TLC	0.037 ± 0.080	0.182 ± 0.158	−4.341	1.16	0.002**

*p < 0.05, **p < 0.01, ***p < 0.001.

Table 5 further presents the statistical analysis of intra-region (ROI–ROI) functional connectivity within the same brain areas, reflecting the intrinsic connectivity strength of local neural networks. The results indicate that the PD group without RAS intervention exhibits significantly higher mean intra-regional connectivity values compared to HC in specific cortical regions (*p* < 0.05). Notably, the TLC demonstrates the most pronounced difference: the PD group has a mean connectivity of 0.292 ± 0.179, significantly greater than the HC group’s 0.133 ± 0.125 (*t* = −3.841, *p* = 0.003, Cohen’s *d =* 1.03). Similarly, the PFC shows enhanced intra-regional connectivity in the PD group (0.274 ± 0.186) compared to HC (0.145 ± 0.112, *t* = −3.147, *p* = 0.009, Cohen’s *d =* 0.84).

**Table 5:** Comparison of intra-regional connectivity in HC group and PD + FOG.

ROI or Ch name	HC	PD + FOG	Statistical value	Cohen’s *d*	*p*-value
PFC-PFC	0.145 ± 0.112	0.274 ± 0.186	−3.147	0.84	0.009**
TLC-TLC	0.133 ± 0.125	0.292 ± 0.179	−3.841	1.03	0.003**

*p < 0.05, **p < 0.01, ***p < 0.001.

##### 3.3.2.2 Comparison of brain network characteristics across intervention groups

**Figure 7:**
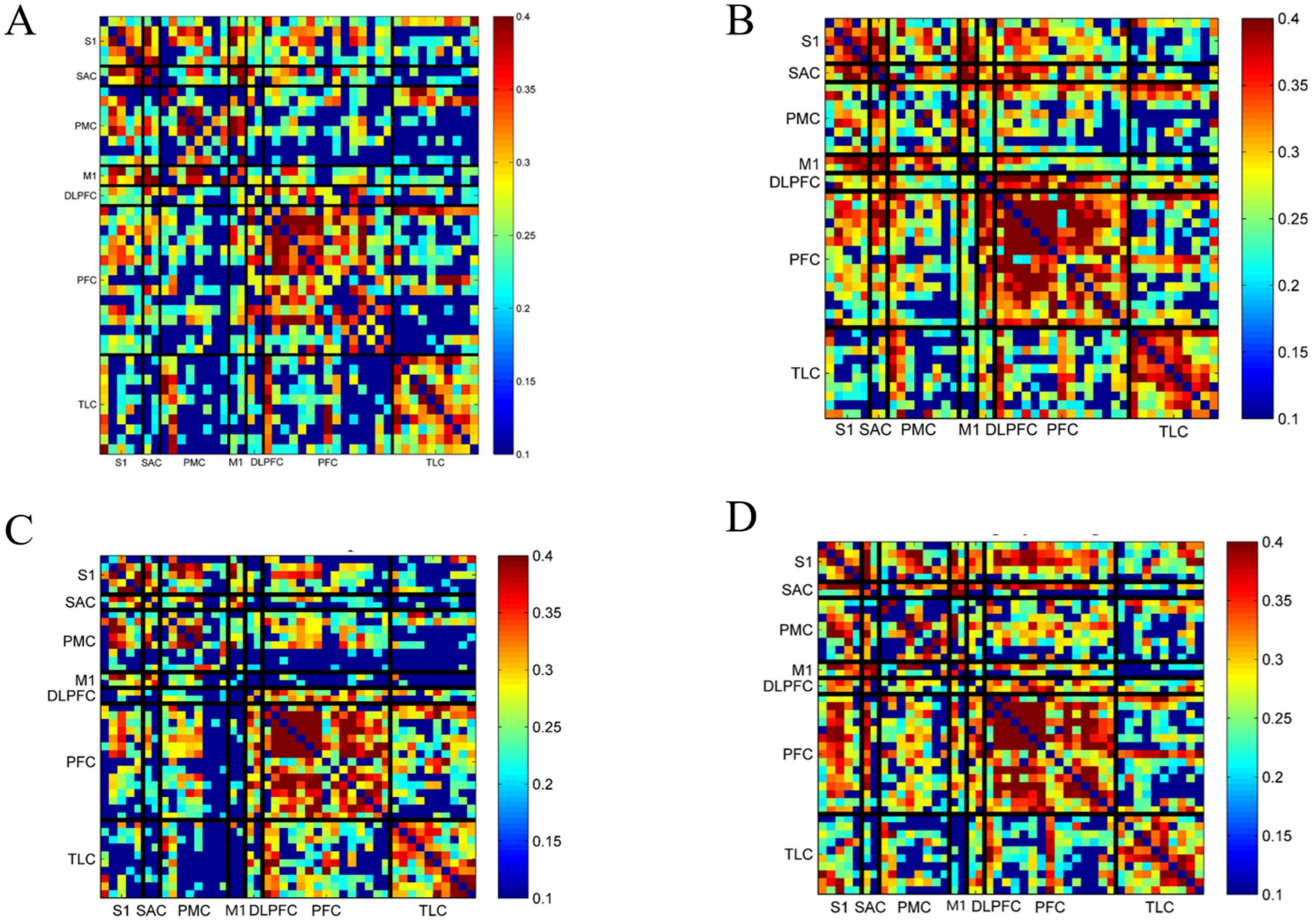
Comparison of functional connectivity across different intervention groups: **(A)** No-intervention group; **(B)** RAS-B group; **(C)** RAS-M group; **(D)** RAS-I group.

As shown in Figure 7, functional connectivity was compared across the four intervention groups. As presented in Table 6, significant intergroup differences in functional connectivity were detected in key cortical connections under different rhythmic auditory stimulation conditions. For channel 1–27 (TLC–PFC), a significant main effect was found (*F* = 3.512, partial *η*^2^ = 0.461, *p* = 0.018), with mean connectivity values of 0.173 ± 0.381 in the None group, 0.320 ± 0.272 in the RAS-B group, 0.263 ± 0.286 in the RAS-M group, and 0.080 ± 0.343 in the RAS-I group; no significant pairwise comparisons were observed. For channel 1–31 (TLC–S1), the main effect reached significance (*F* = 3.023, partial *η*^2^ = 0.498, *p* = 0.033), with values of 0.229 ± 0.341, 0.363 ± 0.370, 0.126 ± 0.340, and 0.100 ± 0.478 in the None, RAS-B, RAS-M, and RAS-I groups, respectively, with no significant post-hoc differences. For channel 3–35 (TLC–PMC), the main effect was significant (*F* = 3.534, partial *η*^2^ = 0.459, *p* = 0.017); connectivity values were 0.239 ± 0.393, 0.153 ± 0.368, −0.086 ± 0.407, and 0.065 ± 0.395 in the None, RAS-B, RAS-M, and RAS-I groups, and *post hoc* tests revealed significantly lower connectivity in the RAS-M group than in the None group (*p* = 0.013). For channel 4–43 (PFC–PMC), a significant main effect was observed (*F* = 3.264, partial *η*^2^ = 0.479, *p* = 0.024), with connectivity values of 0.183 ± 0.284, 0.281 ± 0.316, 0.044 ± 0.313, and 0.088 ± 0.330 across groups, and no significant pairwise differences. For channel 5–42 (TLC–PMC), the main effect was significant (*F* = 3.927, partial *η*^2^ = 0.433, *p* = 0.011), with values of −0.081 ± 0.349, 0.187 ± 0.392, −0.010 ± 0.405, and 0.180 ± 0.296 in the four groups, and no significant pairwise differences. For channel 11–27 (PFC–PFC), a significant main effect was detected (*F* = 4.144, partial *η*^2^ = 0.420, *p* = 0.008), with mean values of 0.189 ± 0.431, 0.384 ± 0.402, 0.526 ± 0.320, and 0.415 ± 0.367; connectivity was significantly higher in both the RAS-B and RAS-I groups than in the None group (*p* = 0.033 and *p* = 0.038, respectively). For channel 16–27 (TLC–PFC), the main effect was significant (*F* = 3.172, partial *η*^2^ = 0.486, *p* = 0.027), with values of 0.279 ± 0.360, 0.187 ± 0.303, 0.254 ± 0.365, and 0.024 ± 0.352; connectivity was significantly higher in the RAS-M group than in the None group (*p* = 0.004). For channel 17–41 (TLC–PMC), a significant main effect was found (*F* = 3.562, partial *η*^2^ = 0.457, *p* = 0.017), with values of 0.196 ± 0.389, 0.181 ± 0.416, −0.042 ± 0.410, and 0.275 ± 0.361; connectivity was significantly higher in the RAS-I group than in the None group (*p* = 0.031). For channel 23–27 (PFC–PFC), the main effect was significant (*F* = 4.681, partial *η*^2^ = 0.390, *p* = 0.004), with connectivity values of 0.034 ± 0.381, 0.325 ± 0.262, 0.347 ± 0.322, and 0.245 ± 0.409, and no significant pairwise differences. For channel 31–35 (S1–PMC), a significant main effect was observed (*F* = 3.225, partial *η*^2^ = 0.482, *p* = 0.025), with values of 0.232 ± 0.371, 0.208 ± 0.324, −0.024 ± 0.414, and 0.246 ± 0.386 across groups, with no significant *post hoc* differences.

**Table 6:** Comparison of key channel functional connectivity across intervention groups.

CH-CH	ROI-ROI	None	RAS-B	RAS-M	RAS-I	Statistical value	Partial *η*^2^	*p*-value	*p*-value (*post hoc*)		
									None vs. RAS-B	None vs. RAS-M	None vs. RAS-I
1–27	TLC-PFC	0.173 ± 0.381	0.320 ± 0.272	0.263 ± 0.286	0.080 ± 0.343	3.512	0.461	0.018	0.216	0.663	0.699
1–31	TLC-S1	0.229 ± 0.341	0.363 ± 0.370	0.126 ± 0.340	0.100 ± 0.478	3.023	0.498	0.033	0.427	0.787	0.598
3–35	TLC-PMC	0.239 ± 0.393	0.153 ± 0.368	−0.086 ± 0.407	0.065 ± 0.395	3.534	0.459	0.017	0.844	0.013	0.336
4–43	PFC-PMC	0.183 ± 0.284	0.281 ± 0.316	0.044 ± 0.313	0.088 ± 0.330	3.264	0.479	0.024	0.981	0.159	0.183
5–42	TLC-PMC	−0.081 ± 0.349	0.187 ± 0.392	−0.010 ± 0.405	0.180 ± 0.296	3.927	0.433	0.011	0.761	0.284	0.570
11–27	PFC-PFC	0.189 ± 0.431	0.384 ± 0.402	0.526 ± 0.320	0.415 ± 0.367	4.144	0.420	0.008	0.033	0.874	0.038
16–27	TLC-PFC	0.279 ± 0.360	0.187 ± 0.303	0.254 ± 0.365	0.024 ± 0.352	3.172	0.486	0.027	0.227	0.004	0.119
17–41	TLC-PMC	0.196 ± 0.389	0.181 ± 0.416	−0.042 ± 0.410	0.275 ± 0.361	3.562	0.457	0.017	0.753	0.993	0.031
23–27	PFC-PFC	0.034 ± 0.381	0.325 ± 0.262	0.347 ± 0.322	0.245 ± 0.409	4.681	0.390	0.004	0.975	0.083	0.934
31–35	S1-PMC	0.232 ± 0.371	0.208 ± 0.324	−0.024 ± 0.414	0.246 ± 0.386	3.225	0.482	0.0250	0.995	0.059	0.999

## 4 Discussion

Previous studies have demonstrated the efficacy of RAS in improving gait disturbances and FOG in PD patients, though the underlying neural mechanisms remain unclear (35). This study systematically investigated the intervention effects and neural mechanisms of three RAS modalities—rhythm listening, music listening, and rhythm imagining—by comparing gait parameters and fNIRS characteristics between the PD + FOG group and the HC group. Results revealed specific gait abnormalities, defective brain region activation, and compensatory brain network characteristics during freezing episodes in PD + FOG patients. They also confirmed that different RAS interventions can improve FOG by targeting functional connectivity within networks centered on the PFC, TLC, and PMC, providing experimental evidence and theoretical support for precise clinical interventions.

### 4.1 Gait characteristics during freezing episodes

This study first identified core gait abnormalities during freezing episodes in PD + FOG patients: compared to normal walking segments, freezing segments exhibited significantly reduced walking speed, shortened stride length, and increased swing width—highly consistent with the clinical phenotype of “motor blockage and gait rigidity” in FOG (36, 37). Further comparison of the intervention effects across three RAS approaches revealed distinct modality-specific effects on gait regulation: Auditory rhythm and music listening significantly improved walking speed and stride length while reducing swing width, and were more effective in reducing FOG episodes. However, clinical observations indicate that music listening intervention substantially increased patient engagement. Imagining rhythms showed some improvement but not significantly. Moreover, the cognitive load generated by internal rhythm production competed for resources with the patients’ existing executive function deficits, limiting the intervention’s effectiveness (38). These findings suggest that externally provided rhythmic cues (listening to beats or music) are more suitable for FOG intervention than internal imagery, as they directly reduce cognitive load and activate sensorimotor neural pathways (39).

### 4.2 Cortical activation in regions of interest

The fNIRS data revealed activation deficits in brain regions of PD + FOG patients: During the freezing phase, HbO_2_ levels in S1, M1, and PFC were significantly higher in the HC group compared to the PD no-intervention group. This phenomenon can be explained by the neurobiological basis of PD: loss of dopaminergic neurons in the brain increases inhibition from the basal ganglia to the motor thalamus, suggesting it may ultimately lead to reduced activation in M1. Deficits in M1 activation directly impact motor execution (40). Previously, the PFC has been extensively studied (41, 42). Consequently, the results show that PD patients exhibit insufficient PFC activation compared to healthy individuals. This finding aligns with prior fMRI studies indicating that reduced activation in motor-related brain regions during PD is directly correlated with dopaminergic neuron degeneration (43–45). However, leveraging the dynamic monitoring advantage of fNIRS, our study further confirms that this activation deficit intensifies specifically during “freezing episodes,” providing neuroimaging evidence for the “context-specific pathological mechanism” of FOG. Following different RAS interventions, mainly TLC showed significant activation. Auditory perception is also a key component of sensory processing, and RAS can synergistically enhance cortical activation in both auditory and motor regions (46).

### 4.3 Functional connectivity between ROIs

Despite reduced activation in localized brain regions, the PD non-intervention group exhibited significantly enhanced functional connectivity within brain networks, spanning both inter-regional and intra-regional dimensions:

Inter-regional connectivity: Connectivity between the sensorimotor area and the TLC was significantly enhanced; connectivity between the prefrontal cortex and motor areas was also markedly elevated. Intra-regional connectivity: Connectivity within the TLC and within the PFC was significantly higher than in the HC group. This study indicates that, compared to healthy individuals, alterations in cortical networks among PD patients primarily manifest as coordinated changes across distinct brain regions. Beyond direct and indirect top-down motor networks for movement control, intracortical networks are also crucial for optimizing resources and ensuring the accuracy of motor output (47). In this study, PD-FOG patients exhibited greater PFC and sensorimotor network connectivity than the HC group. This suggests that in PD-FOG patients, the indirect motor network may be activated and integrated with the direct motor network to control simple tasks, whereas this may not be necessary in HCS or PD-nFOG patients (48, 49). The enhanced intracortical connectivity observed in PD-FOG patients may drive activation of the indirect motor network to counteract the onset of FOG, potentially serving as a compensatory mechanism for impaired motor autonomy in this population.

Substantial evidence indicates that auditory and motor systems are interconnected across multiple levels, including cortical, subcortical, and spinal regions. The auditory system is particularly adept at processing temporal information and connecting with motor brain structures—such as the basal ganglia, M1, SMA, and cerebellum—to synchronize rhythmic signals with motor responses (50, 51). Auditory rhythms serve as external cues that synchronize the motor system, reducing reliance on potentially impaired internal cues. This synchronization helps “constrain” motor patterns, promoting smoother, more rhythmic movement. The RAS is thought to support improved timing and consistency of gait, enhancing overall motor control. Rhythmic auditory stimulation may facilitate motor activation patterns by modulating cortical beta oscillations or enhancing connectivity between frontal, central-parietal, and temporal regions (14). Our FC data indicate that auditory intervention primarily targets the prefrontal and sensorimotor networks: rhythmic beats enhance TLC-PMC connectivity and intra-PFC connections, reinforcing sensorimotor integration and cognitive motor control—consistent with its superior effect on gait improvement. Music listening significantly enhances intra-PFC connectivity (to a greater extent than rhythmic beats), reflecting music’s stronger activation of affective and cognitive networks, which may indirectly support motor persistence. Imagined listening may reduce sensorimotor coupling due to excessive reliance on internal cognition.

PD patients exposed to rhythmic beats exhibited stronger inter-network connectivity between auditory, executive control, and motor networks. This may arise from external rhythmic stimuli drive greater neuronal synchronization. When stimulated by rhythmic beats, auditory brain networks extend to bilateral auditory cortices, demonstrating robust task-related activity (17). Our findings indicate that cortical systems play a crucial role in RAS, with the strongest impact observed in TLC-related networks within the auditory network. In PD patients, connectivity between these networks may help compensate for impaired corticostriatal motor control systems (52). RAS-B and RAS-M primarily enhance the sensorimotor pathways. During music-synchronized movement, the brain must determine the sequence and timing of each action—a neural process requiring high-level integration of action timing and order, leading to heightened activity in the frontoparietal network during movement (53). RAS-related entrainment may influence the frontoparietal control system, amplifying its impact on motor control. This RAS function not only benefits motor task performance but also aligns with research findings. In contrast, internal cue interventions showed relatively stable connectivity patterns.

These findings underscore that externally provided rhythmic cues (beat, music) prove more effective than internal imagery. The PMC plays a crucial role in motor programming and regulation through its extensive cortical networks linking the prefrontal cortex, parietal cortex, and motor cortex (54). Concurrently, the prefrontal cortex plays a central role in integrating auditory rhythms with motor planning. The sensorimotor network (S1, M1) showed no significant activation changes across all intervention groups, suggesting that RAS may primarily exert its effects through higher-order motor control networks (prefrontal cortex, PMC) rather than the primary motor cortex (55).

### 4.4 Limitations

This study has several limitations that should be noted: it only included Parkinson’s disease patients with freezing of gait and healthy controls, lacking a comparison group of PD patients without freezing of gait, which limits the generalizability and interpretability of the findings; each group included only 28 subjects, so future studies should expand sample sizes and incorporate patients across broader disease stages to validate the generalizability of these findings; the experimental design was not counterbalanced, and the fixed task order may have introduced anticipation effects and biases that affected the reliability of the results; functional near-infrared spectroscopy only captures superficial cortical activity and cannot detect deep brain regions such as the basal ganglia and cerebellum that are critical to Parkinson’s disease, and without short-separation channels to correct extracerebral interference, there may be errors in the measured signals; HbO₂ signals were extracted using averaging within task epochs rather than more refined approaches such as GLM-based analysis or time-resolved hemodynamic modeling, which may obscure the temporal dynamics of cortical activation and introduce noise; in addition, the study did not systematically optimize the parameters of rhythmic auditory stimulation, failed to clarify the dose–response relationship between stimulation parameters and intervention effects, and only evaluated the immediate effects of the intervention without long-term follow-up, making it impossible to confirm the sustainability of the intervention effects and the long-term changes in brain network plasticity.

## 5 Conclusion

This study employed fNIRS to investigate changes in brain region activation and functional connectivity during walking tasks with and without RAS in HC and patients with PD + FOG. The following findings were obtained:

(1) Abnormal gait and brain function characteristics in PD + FOG patients: PD + FOG patients exhibited significant gait impairment during freezing episodes, manifested as reduced walking speed and stride length alongside increased swing width. At the neural level, the PD + FOG group exhibited significantly lower activation in sensorimotor cortex (S1, M1) and PFC compared to HC, yet demonstrated enhanced intracortical connectivity. This increased activation within the indirect motor network may represent a compensatory mechanism for impaired motor autonomy in PD-FOG patients.

(2) Intervention efficacy varies across RAS modalities: Auditory beat and music listening yielded more pronounced improvements, whereas auditory imagery showed relatively weaker effects.

(3) Neural mechanisms of RAS in improving FOG: Auditory interventions primarily target the prefrontal cortex and sensorimotor networks. Rhythm listening enhances TLC-PMC connectivity and PFC internal connectivity, strengthening sensorimotor integration and cognitive motor control. Music listening significantly increases PFC internal connectivity (to a greater extent than rhythm listening), reflecting music’s stronger activation of affective and cognitive networks, which may indirectly support motor persistence. Imagination listening may reduce sensorimotor coupling due to excessive reliance on internal cognition.

In summary, this study confirms that distinct RAS improve freezing of gait in Parkinson’s disease patients by specifically modulating functional connectivity between the prefrontal cortex and sensorimotor networks. The underlying neural mechanisms involve rhythm entrainment and brain network remodeling. These findings provide crucial theoretical and experimental support for understanding the pathophysiology of freezing of gait and developing precision intervention strategies.

## Data Availability

The raw data supporting the conclusions of this article will be made available by the authors, without undue reservation.
